# Prevalence of chronic kidney disease and risk factors for its progression: A cross-sectional comparison of Indians living in Indian versus U.S. cities

**DOI:** 10.1371/journal.pone.0173554

**Published:** 2017-03-15

**Authors:** Shuchi Anand, Dimple Kondal, Maria Montez-Rath, Yuanchao Zheng, Roopa Shivashankar, Kalpana Singh, Priti Gupta, Ruby Gupta, Vamadevan S. Ajay, Viswanathan Mohan, Rajendra Pradeepa, Nikhil Tandon, Mohammed K. Ali, K. M. Venkat Narayan, Glenn M. Chertow, Namratha Kandula, Dorairaj Prabhakaran, Alka M. Kanaya

**Affiliations:** 1 Centre for Chronic Conditions and Injuries, Public Health Foundation of India, New Delhi, India; 2 Centre for Chronic Disease Control, New Delhi, India; 3 Division of Nephrology, Stanford University School of Medicine, Palo Alto, CA, United States of America; 4 Madras Diabetes Research Foundation & Dr. Mohan’s Diabetes Specialties Centre, Chennai, India; 5 Department of Endocrinology, All India Institute of Medical Sciences, New Delhi, India; 6 Rollins School of Public Health, Emory University, Atlanta, GA, United States of America; 7 Division of General Internal Medicine and Department of Preventive Medicine, Northwestern University, Chicago, IL, United States of America; 8 Division of General Internal Medicine, University of California San Francisco, San Francisco, CA, United States of America; Peking University First Hospital, CHINA

## Abstract

**Background:**

While data from the latter part of the twentieth century consistently showed that immigrants to high-income countries faced higher cardio-metabolic risk than their counterparts in low- and middle-income countries, urbanization and associated lifestyle changes may be changing these patterns, even for conditions considered to be advanced manifestations of cardio-metabolic disease (e.g., chronic kidney disease [CKD]).

**Methods and findings:**

Using cross-sectional data from the Center for cArdiometabolic Risk Reduction in South Asia (CARRS, n = 5294) and Mediators of Atherosclerosis in South Asians Living in America (MASALA, n = 748) studies, we investigated whether prevalence of CKD is similar among Indians living in Indian and U.S. cities. We compared crude, age-, waist-to-height ratio-, and diabetes- adjusted CKD prevalence difference. Among participants identified to have CKD, we compared management of risk factors for its progression. Overall age-adjusted prevalence of CKD was similar in MASALA (14.0% [95% CI 11.8–16.3]) compared with CARRS (10.8% [95% CI 10.0–11.6]). Among men the prevalence difference was low (prevalence difference 1.8 [95% CI -1.6,5.3]) and remained low after adjustment for age, waist-to-height ratio, and diabetes status (-0.4 [-3.2,2.5]). Adjusted prevalence difference was higher among women (prevalence difference 8.9 [4.8,12.9]), but driven entirely by a higher prevalence of albuminuria among women in MASALA. Severity of CKD—-i.e., degree of albuminuria and proportion of participants with reduced glomerular filtration fraction-—was higher in CARRS for both men and women. Fewer participants with CKD in CARRS were effectively treated. 4% of CARRS versus 51% of MASALA participants with CKD had A1c < 7%; and 7% of CARRS versus 59% of MASALA participants blood pressure < 140/90 mmHg. Our analysis applies only to urban populations. Demographic—-particularly educational attainment—-differences among participants in the two studies are a potential source of bias.

**Conclusions:**

Prevalence of CKD among Indians living in Indian and U.S. cities is similar. Persons with CKD living in Indian cities face higher likelihood of experiencing end-stage renal disease since they have more severe kidney disease and little evidence of risk factor management.

## Introduction

Historically, migration from low- and middle-income countries (LMIC) to high-income countries (HIC) has conferred higher cardio-metabolic risk among the immigrant groups. For example, three West African populations studied along “the migration ladder”—living in Nigeria, Jamaica, and the U.S.—demonstrated higher body mass indices in a stepwise fashion[1]. Indian immigrants to London in the 1990s had higher body mass index, systolic blood pressure, and fasting blood glucose compared with their siblings living in Punjab, India[2]. Japanese immigrants to Hawaii and California in the 1970s experienced a doubling in the incidence of myocardial infarction compared with contemporaries living in Japan[3].

However, as many LMIC experience rapid urbanization—accompanied by a rise in consumption of energy-dense foods and declines in physical activity—it is conceivable that the burden of cardio-metabolic diseases in urban residents of LMIC now approaches that of immigrants to HIC. In fact, recent data suggest that prevalence of diabetes mellitus is *higher* in Indians living in Indian rather U.S. cities [4]. Whether this trend holds true for other cardio-metabolic diseases, particularly ones that are considered late manifestations, is not known.

We therefore compared data from the Center for cArdiometabolic Risk Reduction in South Asia (CARRS) and Mediators of Atherosclerosis in South Asians Living in America (MASALA) studies to: 1. compare the prevalence of chronic kidney disease (CKD) among Indians living in Indian cities (CARRS) with Indians who have immigrated to U.S. cities (MASALA); 2. assess whether differences in body size or diabetes prevalence explain any CKD prevalence difference; and 3. among participants identified to have CKD in the two studies, describe the management of parameters associated with progression to end-stage renal disease (ESRD).

## Methods

The methodologies of the CARRS[5] and MASALA[6] studies have been previously described in detail. Briefly, the CARRS Study is a community-based prospective study that employed a multistage cluster sampling technique to capture the prevalence and incidence of cardio-metabolic diseases in three major cities of South Asia—Chennai and Delhi, India, and Karachi, Pakistan. The study received approval for human subjects research from the Ethics Committees of the Public Health Foundation of India and All India Institute of Medical Sciences (Delhi), Madras Diabetes Research Foundation (Chennai), and Emory University (Atlanta). We restricted this analysis to Delhi and Chennai (n = 12 271) as the laboratory in Karachi used different laboratory kits and equipment for serum and urine creatinine assays. To match MASALA study entry criteria, we further restricted the analysis to participants aged ≥ 40 years old without self-reported heart disease or stroke (n = 6537) **(See Figure A in S1 File for study flowchart)**. Of these, 5294 participants had complete data on albuminuria and serum creatinine, and comprise the analytical group. **Table A in S2 File** demonstrates that while men were more likely to have missing data, participants with and without data on markers of CKD were similar in their age distribution and educational status.

The MASALA study is a prospective study investigating the prevalence and outcomes of subclinical cardiovascular disease in 906 South Asian adults, aged ≥ 40 years and free of physician-diagnosed cardiovascular disease. **(Figure B in S1 File).** This study invited random samples of South Asians (identified as such from census tracts or surrounding counties using surname identification techniques) living in the San Francisco Bay Area and greater Chicago area to participate via mail. Study participants had been living in the U.S. for a mean of 27 ± 11 years. Institutional Review Boards at the University of California, San Francisco and Northwestern University approved the study. In this analysis, we included persons who were born in India (n = 757) and had complete data on albuminuria and serum creatinine (n = 748).

### Correlates of CKD

Both the CARRS and MASALA studies obtained data on age, sex, household income, years of schooling and highest level of education achieved, tobacco use, and use of medications using standardized questionnaires. Both studies also obtained weight, height, waist circumference, and hip circumference measurements using protocols similar to those employed for the U.S. National Health and Nutrition Examination Survey (NHANES). For other demographic and clinical risk factors for CKD and/or progression of CKD, we attempted to harmonize the correlate definitions across the two studies (**Table 1**).

**Table 1:** Harmonizing measures from the CARRS and MASALA studies.

Measure	Methodology in CARRS	Methodology in MASALA	Harmonized measure
**Income**	Monthly household income (categories)	Annual household income (categories)	Compare individuals in top tertile of income versus not
**Physical activity**	International Physical Activity Questionnaire (IPAQ)[37]	Typical Week’s Physical Activity Questionnaire[38]	Weekly vigorous physical activity (yes/no), since both surveys described vigorous physical activity similarly
**Diet**	Frequency of consumption of major food groups for the past year (including typical foods consumed in India)	Study of Health Assessment and Risk in Ethnic Groups (SHARE) questionnaire[39]	Fruit and vegetable intake (number of times/day), since both surveys described these food groups similarly
**Blood pressure**	After 5 minutes of rest, Seated blood pressure at rest at least two times, using an oscillometric device (Omron Dailan Co., Ltd, Dalian, Liaoning, China). A third measurement was obtained if the difference between the first two systolic or diastolic measurements was more than 10 mmHg and 5 mmHg, with at least 30 seconds in between each.	After 5 minutes of rest, seated blood pressure at rest three times with at one minute in between each reading, using an oscillometric device (V100 Vital Signs Monitor, GE Healthcare, Fairfield, CT. U.S.)	Average of last two readings
**Medications**	Obtained 1 year after baseline; individual medication names	Obtained at baseline; individual medication names	Categorized by study investigators into broader categories (yes/no): 1.Blood pressure medications, 2. ACEI/ARB therapy, 3. Anti-glycemic therapy, including insulin, and 4. Lipid therapy, including statins, fibrates and ezetimibe
**Chronic kidney disease**	Spot urine albumin measured via immunoturbidimetric assaySerum and urine creatinine measured via rate-blanked compensated kinetic Jaffe method	Spot urine albumin measured via immunoturbidimetric assay	Creatinine assays in both laboratories traceable to IDMS. CKD defined in both studies as: 1. Single urine albumin to creatinine ratio ≥ 3.4 mg/mmol [30 mg/g], or 2. Single calculation of CKD-EPI eGFR < 60 ml/min/1.73m^2^
		Serum and urine creatinine measured via enzymatic colorometric method	

Abbreviations: IDMS- isotope dilution mass spectrometry; CKD-chronic kidney disease; eGFR-estimated glomerular filtration rate.

### Laboratory measures

Accredited site laboratories processed participants’ fasting blood and urine samples. Both studies employed the same assay methodology for: fasting plasma glucose (hexokinase/kinetic method), glycosylated hemoglobin (high performance liquid chromatography standardized to the National Glycohemoglobin Standardization Program), lipid panel (enzymatic), and urine albumin (immunoturbidimetric). To measure urine and serum creatinine, CARRS used the rate-blanked and compensated kinetic Jaffe assay whereas MASALA used the enzymatic colorometric assay, both traceable to isotope dilution mass spectrometry (IDMS) at the National Institute of Standards[7]. The two assays have been shown to have a nearly identical reference range [8–10].

### Definitions of disease status

With the 2012 Kidney Disease Improving Global Outcomes (KDIGO) guidelines[11] as a reference, we defined a participant as having CKD with albuminuria (albumin-to-creatinine ratio ≥ 3.4 mg/mmol [30 mg/g]) and/or CKD-EPI[12] estimated glomerular filtration rate (eGFR) < 60 ml/min/1.73m^2^. We defined diabetes as fasting glucose ≥ 7 mmol/L (126 mg/dL) and/or use of medications for diabetes; and hypertension as systolic BP ≥ 140 or diastolic ≥ 90 mmHg and/or use of medications for hypertension.

### Statistical analyses

Using means and standard deviations for continuous, or counts and percentages for categorical variables, we described baseline characteristics stratified by sex and study type. We report raw, age-adjusted, and sex-stratified prevalence of overall CKD, eGFR < 60 ml/min/1.73m^2^, and albuminuria in each study. We also examined prevalence of CKD according to the following demographic and behavioral categories: income, education, physical activity, and fruit and vegetable intake.

We adjusted the between-study prevalence difference in overall CKD, eGFR < 60 ml/min/1.73m^2^, and albuminuria for age, waist-to-hip ratio, and diabetes, using a generalized linear model with a log link and Binomial distribution (log-Binomial), or Poisson with robust standard errors (modified Poisson model) if the log-Binomial model failed to converge[13]. While persons with vascular disease can manifest hypertension and CKD, hypertension is also often a consequence of CKD. We therefore did *not* adjust the prevalence difference in CKD for prevalence of hypertension in the two studies.

Since overall missingness in the CARRS analytic study was approximately 20%, we performed multiple imputation for missing covariates[14]. We assumed data to be missing at random and used the Fully Conditioning Specification[15] approach to impute 20 full datasets, stratified by sex. The MASALA study had only two missing observations; we thus performed a complete case analysis for this study.

In the participants with CKD in the two studies, we describe differences in prevalence of risk factors associated with progression to ESRD and/or cardiovascular events in the two studies. Further, we estimate the relative likelihood of an important clinical outcome—i.e., ESRD or death due to kidney disease—among participants with diabetes and CKD in CARRS versus MASALA. We used recently described five-year event rates from the standard arm of the multi-country Action in Diabetes and Vascular Disease: Preterax and Diamicron MR Controlled Evaluation (ADVANCE) study[16]. We obtained these event rates stratified according to A1c category (<8 versus ≥ 8%), and multiplied the proportion of CKD participants falling in the these two A1c categories with the respective events rates to estimate a relative risk in CARRS versus MASALA. We used SAS, version 9.4 (SAS Institute, Inc., Cary, NC) or Stata version 13.1 (StataCorp. 2013. Stata Statistical Software: Release 13. College Station, TX: StataCorp LP.) to perform all analyses.

## Results

Table 2 provides details of participant characteristics in the CARRS and MASALA studies, stratified by sex. In general, CARRS participants were younger, with substantially fewer men and women in the ≥ 55 years of age categories. Educational attainment was strikingly different between the two studies: more than 85% of men and women in the MASALA study had attained a college degree, whereas fewer than 20% in CARRS had done so. Close to a quarter of participants had diabetes, with the exception of women in the MASALA study in whom the prevalence was 15%. More women in both studies had abnormal waist-to-height ratio; more men in both studies reported smoking and performing vigorous physical activity.

**Table 2:** Demographic, anthropometric, laboratory and disease status in the CARRS and MASALA studies, 2010–2013.

	Men		Women	
	CARRSN = 2563	MASALA N = 402	CARRS N = 2731	MASALA N = 346
**Demographics**				
Mean age, years	51.9 ± 9.8	56.2 ± 9.9	50.9 ± 8.9	54.6 ± 8.6
40 to 54	1705 (66.5)	191 (47.5)	1878 (68.8)	186 (53.8)
55 to 69	681 (26.6)	164 (40.8)	732 (26.8)	142 (41.0)
≥70	177 (6.9)	47 (11.7)	121 (4.4)	18 (5.2)
Less than college degree	2045 (79.8)	30 (7.5)	2367 (86.7)	44 (12.7)
Current tobacco user*	772 (30.1)	24 (6.0)	21 (0.8)	4 (1.2)
Missing	-	1 (0.2)	-	-
Fruits and Vegetable Intake (# of times/day)				
<2	1063 (41.5)	11 (2.7)	1383 (50.6)	2 (0.6)
2 to 4	1244 (48.5)	68 (16.9)	1173 (43.0)	28 (8.1)
>4	256 (10.0)	322 (80.1)	175 (6.4)	316 (91.3)
Missing	-	1 (0.2)	-	-
Any vigorous physical activity in the week	450 (17.6)	120 (29.9)	271 (9.9)	54 (15.6)
Missing	17 (0.7)	13 (3.2)	21 (0.8)	3 (0.9)
**BP and anthropometry**				
Mean waist-to-height ratio	0.55 ± 0.07	0.57 ± 0.06	0.57 ± 0.08	0.57 ± 0.06
Abnormal waist-to-height ratio^¶^	1541 (60.1)	353 (87.8)	1871 (68.5)	299 (86.4)
Missing	560 (21.8)	2 (0.5)	491 (18.0)	-
BP(mmHg): Sys &/or Dias^†^				
<120 & <80	535 (20.9)	141 (35.1)	769 (28.2)	169 (48.8)
120–139 or 80–89	958 (37.4)	189 (47.0)	1029 (37.7)	117 (33.8)
≥140 or ≥90	910 (35.5)	72 (17.9)	859 (31.5)	60 (17.3)
Missing	160 (6.2)	-	74 (2.7)	-
**Laboratories**^†^				
Fasting glucose (mmol/L)				
< 5.6	1196 (46.7)	200 (49.8)	1149 (42.1)	235 (67.9)
5.6 to <7	798 (31.1)	138 (34.3)	1026 (37.6)	92 (26.6)
≥7	568 (22.2)	58 (14.4)	555 (20.3)	18 (5.2)
Missing	1 (0.0)	6 (1.5)	1 (0.0)	1 (0.3)
Hemoglobin A1c (%)				
<5.7	649 (25.3)	115 (28.6)	578 (21.2)	98 (28.3)
5.7 - <6.5	993 (38.7)	202 (50.2)	1126 (41.2)	206 (59.5)
≥6.5	908 (35.4)	83 (20.6)	1006 (36.8)	40 (11.6)
Missing	13 (0.5)	2 (0.5)	21 (0.8)	2 (0.6)
Diabetes^#^	624 (24.3)	97 (24.1)	631 (23.1)	52 (15.0)
Missing	1 (0.0)	-	1 (0.0)	-
Hypertension^ǁ^	987 (38.5)	181 (45.0)	971 (35.6)	126 (36.4)
Missing	154 (6.0)	-	63 (2.3)	-

Data are expressed as mean (standard deviation), or number (percent in each group).

*Current tobacco use is defined as any cigarette use in the past 12 months.

^¶^Waist-to-height ratio > 0.5 is defined as abnormal

^†^Blood pressure and laboratory values report measured results regardless of self-reported disease status.

^#^Diabetes is defined as fasting glucose ≥ 7 mmol/L (126 mg/dL) and/or use of medications for diabetes.

^ǁ^Hypertension is defined as systolic BP ≥ 140 or diastolic ≥ 90 mmHg and/or use of medications for hypertension.

### Prevalence of CKD

Raw and age-adjusted prevalence of overall CKD was similar among men in CARRS and MASALA, but substantially higher among women in MASALA compared with women in CARRS (**Table 3**). Prevalence increased with age in both studies, and for both sexes (**See Table B in S2 File for age- and sex- stratified CKD prevalence**). Overall, there was a modestly higher prevalence of CKD in MASALA (14.0% [95% CI 11.8–16.3]) than in CARRS (10.8% [95% CI 10.0–11.6]).

**Table 3:** Prevalence of chronic kidney disease in the CARRS and MASALA studies.

* *	Overall		Men		Women	
* *	CARRS	MASALA	CARRS	MASALA	CARRS	MASALA
	N = 5294	N = 748	N = 2563	N = 402	N = 2731	N = 346
**Raw Prevalence**						
CKD	10.5 (9.7–11.4)	16.3 (13.7–19.0)^+^	10.5 (9.3–11.7)	12.2 (9.0–15.4)	10.6 (9.4–11.7)	21.1 (16.8–25.4)^+^
Albuminuria	8.6 (7.9–9.4)	15.1 (12.5–17.7)^+^	8.5 (7.4–9.5)	10.9 (7.9–14.0)	8.8 (7.7–9.8)	19.9 (15.7–24.2)^+^
eGFR<60	3.1 (2.6–3.6)	2.0 (1.0–3.0)	3.0 (2.3–3.7)	2.2 (0.8–3.7)	3.2 (2.5–3.8)	1.7 (0.4–3.1)
**Age-adjusted Prevalence**						
CKD	10.8 (10.0–11.6)	14.0 (11.8–16.3)^+^	10.8 (9.6–12.0)	10.3 (7.6–13.0)	10.8 (9.6–11.9)	18.6 (14.9–22.4)^+^
Albuminuria	8.7 (8.0–9.5)	13.8 (11.4–16.1)^+^	8.6 (7.5–9.7)	9.8 (7.1–12.6)	8.8 (7.7–9.9)	18.5 (14.5–22.4)^+^
eGFR<60	3.2 (2.8–3.7)	1.6 (0.8–2.3) ^+^	3.1 (2.5–3.8)	1.7 (0.6–2.8)^+^	3.2 (2.6–3.9)	1.4 (0.3–2.5)^+^

Values in table are prevalence % (95% confidence interval).

^+^p value for prevalence difference from CARRS < 0.05

Investigating albuminuria further we found that while the *prevalence* of albuminuria was higher in MASALA than in CARRS, the *severity* of albuminuria was higher in CARRS: log-mean albuminuria value was 4.5 ± 1.0 versus 4.1 ± 0.8 (p value = 0.01) among women in CARRS and MASALA respectively, and 4.6 ± 1.2 versus 4.2 ± 0.7 (p value < 0.001) among men in CARRS and MASALA (**See Figures C-E in S1 File for albuminuria distribution and Table C in S2 File for albuminuria categories**). Odds of albuminuria did not differ by meat intake status in either study (data not shown).

**Fig 1:**
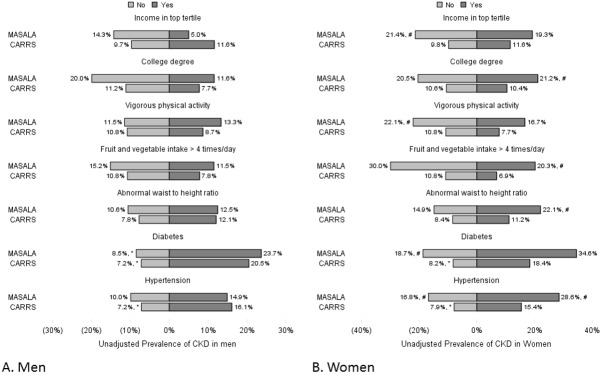
**Prevalence of CKD according to demographic correlates in (A) men and (B) women** In MASALA, men with income in the lower tertiles had higher CKD prevalence than men with income in the top tertile. In CARRS, men with no college education had higher CKD prevalence than men with college education. Across studies, men with income in the lower tertiles in CARRS had higher CKD prevalence than men with income in the lower tertiles in the MASALA. Women in the MASALA study had significantly higher prevalence of CKD across nearly all demographic correlates compared with women in CARRS. * denotes statistically significant difference within each study, # denotes statistically significant difference between studies.

For both men and women, prevalence of CKD was higher in the highest tertile of income in CARRS, but lower in MASALA when compared with participants in the lower tertiles of income (**Fig 1A and 1B**). For other potential correlates such as physical activity, diabetes, and hypertension, CARRS and MASALA study participants had similar directions of associations with CKD. Women in MASALA had higher prevalence of CKD than women in CARRS across all correlates.

### Adjusted prevalence difference in CKD

**Fig 2:**
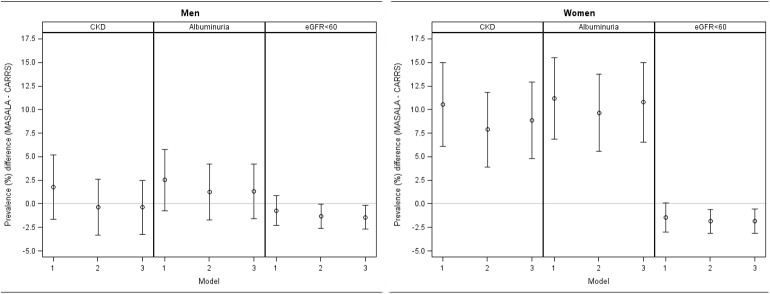
**Prevalence difference in CKD in the MASALA study from the CARRS study (A) men and (B) women.** We present prevalence difference in CKD 1. Unadjusted, 2. Adjusted for age, and 3. Adjusted for age, waist-to-height ratio, and diabetes. Prevalence difference in overall CKD and albuminuria among men in CARRS and MASALA was negligible in all three models; prevalence of eGFR < 60 ml/min/1.73m^2^ was slightly higher in men in CARRS. Unadjusted prevalence in overall CKD and albuminuria among women in MASALA was 11.1% and 11.8% higher respectively compared with CARRS; adjusting for diabetes and waist-to-height ratio did not attenuate this prevalence difference.

We examined the prevalence difference in CKD after adjusting for diabetes, waist-to-height ratio, and the residual effects of age (**Fig 2A and 2B**). Adjustment for these covariates led to a slight attenuation in the magnitude of the CKD prevalence difference between MASALA and CARRS for both men and women. Nonetheless, the main findings remained unchanged. Men in the two studies had similar prevalence of CKD, with albuminuria prevalence higher in MASALA and eGFR < 60 ml/min/1.73m^2^ prevalence higher in CARRS. Women in MASALA had a substantially higher prevalence of overall CKD and albuminuria than women in CARRS, but the prevalence of eGFR < 60 ml/min/1.73m^2^ was slightly higher in women in CARRS.

In sensitivity analyses adjustment for hypertension status led to a further slight attenuation of the prevalence difference between women (**Table D in S2 File**). Since income had a differential relationship with CKD in the two studies, we performed stratified analyses further adjusting for income and education. Among participants in the top tertile of income, men in MASALA seemed to have slightly *lower* CKD prevalence, whereas women in MASALA continued to demonstrate a higher CKD prevalence compared with counterparts in CARRS.

### Risk factor management in participants with CKD

**Fig 3:**
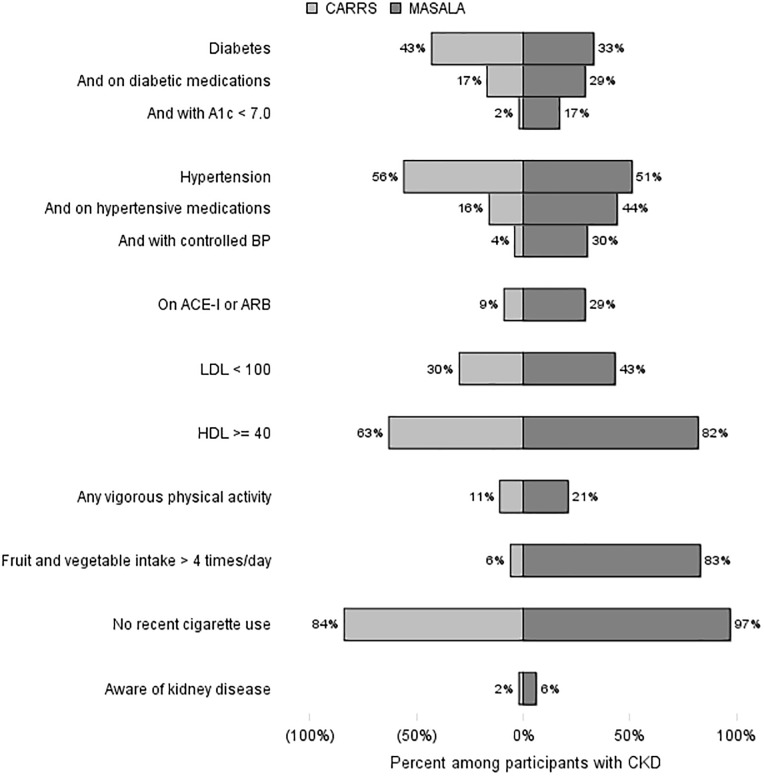
Prevalence of risk factors for adverse events, and evidence of their management among participants with CKD. Of the 558 and 122 participants with CKD in CARRS and MASALA respectively, 430 (77%) and 119 (98%) had complete data on prevalence of risk factors for progression of CKD and/or cardiovascular events. While 43% of participants with CKD in CARRS had diabetes, only 17% were on medications and only 2% (i.e., 4% of those with CKD and diabetes) had A1c < 7.0.

Among participants identified to have CKD in the two studies, the prevalence of hypertension was similar and the prevalence of diabetes was lower in the MASALA than in the CARRS study (**Fig 3**). Fewer participants in CARRS with these conditions were treated with medications and fewer had evidence of meeting targets such as hemoglobin A1c < 7.0 among those with diabetes (4% in CARRS versus 51% in MASALA) or blood pressure < 140/90 mmHg (7% in CARRS versus 59% in MASALA) among those with hypertension. In sensitivity analyses, we restricted this comparison to patients with a college degree or more, or to patients in the top tertile of income and found that while the likelihood of meeting targets went up in both groups, the gap between CARRS and MASALA participants in meeting targets remained large. For example, among those with diabetes and CKD in the top income tertile, 9% of CARRS participants had A1c < 7.0 compared with 63% in MASALA.

Since event rates for ESRD and/or death due to kidney disease are more than two-times higher among those with poor glycemic control (≥ 8%) and CKD[17], CARRS participants with diabetes and CKD are estimated to have 40% higher risk for experiencing this combined outcome (relative risk 1.4, 95% CI: 0.8–2.6).

Behavioral characteristics known to attenuate risk for progression of CKD (e.g., physical activity and abstaining from tobacco use) and/or associated cardiovascular events (e.g., fruit and vegetable intake) were also more likely to be suboptimal in the CARRS than in MASALA study. Awareness of presence of CKD was low in both studies.

## Discussion

Our study finds that the overall age-adjusted prevalence of CKD in Indians living in Indian cities approaches that of Indians living in U.S. cities. Men in particular provided the strongest evidence for a change in the trend that migration to HIC results in a dramatic increase in risk for cardio-metabolic diseases. Furthermore, compared with counterparts living in the U.S., those with CKD living in urban India have more severe CKD and worse risk factor profiles—e.g., higher likelihood of uncontrolled A1c or untreated hypertension—rendering them vulnerable to experiencing more cardiovascular events and rapid kidney disease progression.

Women participating in the MASALA study had a substantially higher prevalence of albuminuria without reduction in eGFR than women participating in CARRS. The small prevalence difference in overall CKD between the two studies was driven in its entirety by a substantially higher prevalence of albuminuria without a reduction in eGFR among women participating in MASALA. Counter-intuitively, women in MASALA had similar prevalence of hypertension, and lower prevalence of diabetes. They also attained higher educational status, performed more physical activity, and consumed more fruits and vegetables than women in CARRS. Odds of albuminuria did not vary by meat intake status in the two studies. Women in MASALA did have higher prevalence of abnormal waist-to-height ratio compared with women in CARRS. Studies have linked central obesity to the presence[18] and severity[19] of albuminuria. In 205 South Asian adults without diabetes, the odds of albuminuria were 4-fold higher in the group with highest waist-to-hip ratios, despite accounting for age, smoking status, and blood pressure[20]. However, in our study, the albuminuria prevalence difference among women persisted even after adjusting for age, diabetes, and waist-to-height ratio. Since a single measure indicating presence of albuminuria strongly predicts 24 hour urine collection results[21], as well as future cardiovascular[22] and renal[23] outcomes, this finding deserves further exploration.

Demographic correlates had a similar direction of association with prevalence of CKD in CARRS and MASALA, with the notable exception of income. Compared with participants in the highest income tertile, those with lower incomes were more likely to have CKD in MASALA; the reverse was true for CARRS. A majority of studies in the U.S. and other HIC have shown that higher income and educational status are associated with lower likelihood of chronic diseases[24]. This pattern has not yet emerged in LMIC, where higher socioeconomic groups experience higher metabolic risk. Lower socioeconomic groups are more likely facing restricted caloric intake and/or physically active in their jobs, thereby counterbalancing other high risk behaviors such as tobacco use and low fruit and vegetable intake[25, 26].

Participants with CKD in the CARRS study demonstrated more severe kidney disease, and most were not effectively managing risk factors. When restricting to participants with CKD who also had a college degree or were in the top tertile of income–so a cohort more similar to the MASALA participants–the proportion of patients on medications increased but a vast majority (90% or more with diabetes or hypertension) were not meeting targets. Clearly a lack of awareness of their condition[27, 28] is a major reason, but even among those with a diagnosis of diabetes or hypertension, there is little understanding of the need for regular medical care[29]. In a large survey performed in Chennai, only 40% of patients with diabetes knew that their disease could lead to any organ complications[30]. Many experts also point to ‘clinical inertia’ in initiating and titrating medications[31, 32]. In an international comparison of physicians practices in managing diabetes, Indian physicians were among the most likely to delay insulin therapy[33].

The lack of glycemic and blood pressure control has important implications for consequent ESRD. Follow up data from the ADVANCE trial—which recruited participants from India, China, and Eastern European countries in addition to HIC—demonstrate that hemoglobin A1c can serve as a universal and significant predictor of ESRD[17]. If we apply the event rates from the standard arm follow up of this trial, CARRS participants with diabetes and CKD are at 40% higher risk of experiencing ESRD or death due to kidney disease, since a much larger proportion of them currently have hemoglobin A1c ≥ 8%. On the other hand, the ADVANCE trial also proves that aggressively treating these risk factors can mitigate the most serious risks associated with CKD in persons from a range of ethnicities, living in settings with a range of healthcare resources [34].

Even among highly educated participants of the MASALA study (nearly all of whom avoid smoking and eat fruits and vegetables regularly), we identified significant gaps in meeting targets for diabetes or hypertension management in the participants with CKD. Similar gaps are noted in the rest of the U.S. Using data from the 2005–2010 NHANES, the United States Renal Data System reports that 48% of participants with CKD and diabetes had A1c < 7.0%; 51% meet this target in MASALA[35]. About a third of participants with CKD in our study and in NHANES 2005–2010 [36] have been prescribed angiotensin converting enzyme inhibitors or angiotensin II receptor blockers.

Our study has several strengths. First, since a majority of participants in the MASALA study were born in India, we were able to test the impact of “residence” (i.e., U.S. metropolitan versus Indian metropolitan areas) in genetically similar populations. Second, both studies used standardized and comparable methodologies for laboratory, blood pressure, and anthropometric ascertainment. Because both albuminuria and IDMS-standardized serum creatinine were measured in the studies, we were able to use the most widely-accepted definition of CKD. Detailed ascertainment of medication use allowed us to compare management of risk factors among participants with CKD.

Limitations of our study include the different eligibility criteria and sampling techniques used in CARRS and MASALA, and while we attempted to select a subset of CARRS cohort to match MASALA entry criteria, important demographic differences in the two studies’ participants remain a source of bias. Most strikingly and as reflective of South Asian immigrants to the U.S., the MASALA participants attained a much higher level of education than CARRS participants. Serum creatinine was also measured via two different assays in the two studies, but the two have been shown to have excellent agreement[10] and were calibrated against the same standard (IDMS)[7], further minimizing inter-assay variation. Both studies only assessed serum creatinine and urine albumin to creatinine at a single time point, and both may therefore be over-estimating the prevalence of CKD since we cannot assess for persistence of abnormal results. Since we studied only urban populations, we cannot generalize to the entire populations of Indians living in either region. Finally, the overall nature of our cross-sectional analyses is descriptive, without ability to draw causal inferences.

In conclusion, the prevalence of CKD in Indians living in Indian and U.S. cities is similar. When we compare the risk profile of individuals with CKD, it is evident that those living in Indian cities are substantially more likely to face worse outcomes. As more and more people living in LMIC move to urban settings, their likelihood of disease may be similar, but likelihood of receiving effective treatment is much lower than counterparts living in HIC. Focused and contextually-appropriate programs targeting aggressive metabolic control can help close these gaps.

## Supporting information

S1 File Figures A-B: Study flowcharts demonstrating creation of analytic group. Figures C-E: Distribution of log-albumin to creatinine ratio in CARRS versus MASALA participants with albuminuria.
(DOCX)

S2 File Table A. Age and education in participants with and without available information on albuminuria and serum creatinine in the CARRS* study. Table B. Age-stratified CKD prevalence in CARRS and MASALA studies. Table C: Albuminuria in the CARRS and MASALA studies. Table D: CKD prevalence (%) difference, after adjustment.
(DOCX)
