# Supplementary material for: Prevalence of chronic kidney disease and risk factors for its progression: A cross-sectional comparison of Indians living in Indian versus U.S. cities
Source: PLoS One. 2017 Mar 15;12(3):e0173554. doi: 10.1371/journal.pone.0173554 (PMC5351850; doi:10.1371/journal.pone.0173554)
Supplement: S1 File — Figures A-B: Study flowcharts demonstrating creation of analytic group. Figures C-E: Distribution of log-albumin to creatinine ratio in CARRS versus MASALA participants with albuminuria. (DOCX) [file pone.0173554.s001.docx]

**S1 Figure A-B: Selection of analytic group from CARRS and MASALA**

1. **Analytic group CARRS**
2. **Analytic group MASALA**

**S1 C-E**: Distribution of log-albumin to creatinine ratio in CARRS versus MASALA participants with albuminuria

1. Overall

1. Men

1. Women
