# Supplementary material for: Prevalence of chronic kidney disease and risk factors for its progression: A cross-sectional comparison of Indians living in Indian versus U.S. cities
Source: PLoS One. 2017 Mar 15;12(3):e0173554. doi: 10.1371/journal.pone.0173554 (PMC5351850; doi:10.1371/journal.pone.0173554)
Supplement: S2 File — Table A. Age and education in participants with and without available information on albuminuria and serum creatinine in the CARRS* study. Table B. Age-stratified CKD prevalence in CARRS and MASALA studies. Table C: Albuminuria in the CARRS and MASALA studies. Table D: CKD prevalence (%) difference, after adjustment. (DOCX) [file pone.0173554.s002.docx]

**S2 FILE:**

**Table A. Age and education in participants with and without available information on albuminuria and serum creatinine in the CARRS* study**

|  | **Men** | | **Women** | |
| --- | --- | --- | --- | --- |
|  | CKD data available N=2563 | CKD data unavailable N=667 | CKD data available  N=2731 | CKD data unavailable N=576 |
| **Demographics** | | | | |
| Mean age, years | 51.9±9.8 | 52.4±10.2 | 50.9±8.9 | 51.9±9.8 |
| 40 to 54 | 1705 (66.5) | 428(64.2) | 1878(68.8) | 361 (62.7) |
| 55 to 69 | 681(26.6) | 191(28.6) | 732(26.8) | 172 (29.9) |
| ≥70 | 177(6.9) | 48(7.2) | 121(4.4) | 43 (7.5) |
| Less than college degree | 2045 (79.8) | 553 (82.9) | 2367 (86.7) | 493 (85.6) |

*Analysis restricted to persons without self-reported heart disease or stroke, and age ≥ 40 years.

**Table B. Age-stratified CKD prevalence in CARRS and MASALA studies**

|  | Overall | | Men | | Women | |
| --- | --- | --- | --- | --- | --- | --- |
|  | CARRS N=5294 | MASALA N=748 | CARRS N=2563 | MASALA N=402 | CARRS N=2731 | MASALA N=346 |
| CKD | 558(10.5) | 122(16.3) | 269(10.5) | 49(12.2) | 289(10.6) | 73(21.1) |
| Age category (years)  40 to 54  55 to 69  ≥ 70 | 286 (8.0)  178 (12.6)  94 (31.5) | 51 (13.5)  54 (17.7)  17 (26.2) | 129 (7.6)  78 (11.5)  62 (35.0) | 16 (8.4)  22 (13.4)  11 (23.4) | 157 (8.4)  100 (13.7)  32 (26.5) | 35 (18.8)  32 (22.5)  6 (33.3) |

**() represent crude CKD prevalence for that age group**

**Table C: Albuminuria in the CARRS and MASALA studies.**

| **Albuminuria category (mg/g)** | Men in CARRS  N (%) | Men in MASALA  N (%) | Women in CARRS  N (%) | Women in MASALA  N (%) |
| --- | --- | --- | --- | --- |
| **0-30** | 2346 (91.5) | 358 (89.1) | 2492 (91.3) | 278 (80.3) |
| **≥30-300** | 185 (7.2) | 43 (10.7%) | 208 (7.6) | 64 (18.5) |
| **≥300** | 32 (1.3) | 1 (0.2%) | 31 (1.1) | 4 (1.2) |

**Table D: CKD prevalence (%) difference, after adjustment**

| ***Sex*** | ***Outcome*** | ***Model1*** | ***Model2*** | ***Model3*** | ***Model4a*** | ***Model4b*** |
| --- | --- | --- | --- | --- | --- | --- |
| Men | CKD | 1.8(-1.6,5.3) | -0.4(-3.2,2.5) | -1.0(-3.8,1.7) | -2.4(-9.9,5.0) | 5.4(-3.3,14.0) |
| Men | Albuminuria | 2.6(-0.7,5.8) | 1.3(-1.6,4.2) | 0.8(-2.0,3.6) | 1.8(-7.0,10.6) | 7.7(-1.3,16.7) |
| Men | eGFR<60 | -0.7(-2.3,0.9) | -1.4(-2.7,-0.2) | -1.7(-2.9,-0.5) | -3.2(-5.6,-0.8) | -0.7(-2.9,1.5) |
| Women | CKD | 10.5(6.1,15.0) | 8.9(4.8,12.9) | 8.7(4.7,12.7) | 10.0(0.9,19.1) | 6.4(-1.5,14.4) |
| Women | Albuminuria | 11.2(6.8,15.5) | 10.8(6.5,15.0) | 10.6(6.4,14.8) | 12.8(3.0,22.6) | 11.6(1.3,21.8) |
| Women | eGFR<60 | -1.5(-3.0,0.1) | -1.8(-3.1,-0.6) | -1.9(-3.1,-0.6) | -3.4(-5.4,-1.4) | -1.0(-3.4,1.4) |

Outcomes are absolute prevalence (%) difference (MASALA - CARRS)

- Model 1 is unadjusted
- Model 2 adjusts for age, waist-to-height ratio, diabetes
- Model 3 adjusts for age, waist-to-height ratio, diabetes, hypertension
- Models 4a and 4b adjust for age, waist-to-height ratio, diabetes, hypertension, education, income, but are stratified by income category. In in model 4a participants with income in the top tertile are included; the remaining participants are in Model 4b.
